# Editing the trajectory of hypertrophic cardiomyopathy

**DOI:** 10.20517/jca.2023.19

**Published:** 2023-06-16

**Authors:** Mason E. Sweat, William T. Pu

**Affiliations:** 1 Departmnet of Cardiology, Boston Children’s Hospital, Boston, MA 02115, USA; 2 Harvard Stem Cell Institute, Harvard University, Cambridge, MA 02138, USA

The genetic code can be coldly tyrannical when it leads a single nucleotide change to alter an individual’s life trajectory. In hypertrophic cardiomyopathy (HCM), dominant pathogenic variants (PVs) in sarcomere genes cause ventricular muscle thickening, hypercontractility, diastolic dysfunction, cardiac fibrosis, and the risk of life-threatening arrhythmias. With a prevalence as high as 1 in 500 individuals^[1]^, HCM imposes considerable medical and economic costs. Despite advances in genetic diagnosis and an improved understanding of its molecular pathogenesis, HCM remains incurable and can progress to heart failure, cardiac transplantation, and premature death. Although small molecules that target HCM’s underlying pathogenic mechanisms have begun to enter clinical use^[2]^, it is likely that cures will require therapies that correct the underlying genetic lesions. The advent of efficient gene editing technologies has opened the door to therapies that correct causative HCM variants. Recent studies published in the February 2023 issue of *Nature Medicine* by the Olson (Chai *et al.*^[3]^) and Seidman (Reichart *et al.*^[4]^) groups have established proof-of-concept that precise and efficient gene editing can be achieved in postnatal mammalian cardiomyocytes and prevent HCM in experimental disease models.

Dominant PVs in myosin heavy chain *7* (*MYH7*), the major myosin isoform in human ventricles, cause approximately 30% of HCM. Among over 250 *MYH7* variants that cause HCM, one of the more common is c.1208G>A, which substitutes glutamine for arginine at protein position 403 (*MYH7*^*R403Q*^). In mice, *Myh6* rather than *Myh7* is the major myosin isoform in mature cardiomyocytes, and *Myh6*^*R403Q/+*^ mice are a well-studied model of HCM^[5]^. Recently, adenine base editors (ABEs) have been developed that allow precise conversion of A to G^[6]^, suggesting that they could be used to correct the c.1208G>A genetic lesion. ABEs comprise a Cas9 enzyme, modified to introduce single-strand nicks, fused to an engineered deaminase that converts adenine to inosine^[6]^ [Figure 1]. When the ABE is directed to a target by a sequence-specific guide RNA (gRNA), the ABE converts an adenine within a restricted “editing window” in the target sequence to inosine and at the same time nicks the unmodified DNA strand. The nick stimulates the cells’ DNA repair machinery, ultimately installing G in place of A. Several different ABE variants with site-specific differences in efficiency and specificity have been developed. The Seidman and Olson groups each selected a different ABE variant for their proof-of-concept *in vivo* efficacy studies, at least in part because the Seidman group focused on the murine *Myh6*^*R403Q*^ target sequence and the Olson group focused on the human *MYH7*^*R403Q*^ target sequence.

The Olson group started by testing different ABEs and a gRNA designed to target the *MYH7*^*R403Q*^ allele in human induced pluripotent stem cells (iPSCs) and iPSC-derived cardiomyocytes (hiPSC-CMs)^[3]^. They demonstrated highly efficient (> 98%) on-target editing in cells efficiently transduced with all editing components, and minimal editing at computationally predicted off-target sites. As expected, gene editing corrected HCM phenotypes observed in *MYH7*^*R403Q/+*^ iPSC-CMs. Next, to test the efficacy of the human sequence-specific editing system *in vivo*, the authors generated a humanized HCM mouse model in which sequences flanking the human *MYH7*^*R403Q*^ variant replace the native *Myh6* murine sequence (*Myh6^h403^*). Although identical at the amino acid level, the encoding DNA sequences differ. The wild-type *Myh6* allele retained the native murine sequence. This strategy enabled the on-target activity of human-specific gRNAs to be tested in mice. However, it precluded the evaluation of *in vivo* off-target editing of the wild-type allele. To deliver the ABE to postnatal cardiomyocytes, the authors used cardiotropic adeno-associated virus 9 (AAV9) and the cardiac-selective cardiac troponin T promoter to express the ABE. The limited cargo capacity of AAV9 mandated a dual AAV strategy, in which the ABE was split across two AAVs and spliced together using trans-splicing inteins^[7]^. Additionally, each of the two AAVs contained a gRNA expression cassette. Delivery of the dual AAV9 ABE system by intrathoracic injection of neonatal pups with up to 2E14 vg/kg (nearly the highest dose administered in clinical trials) eliminated detectable HCM phenotypic manifestations at 16 weeks in an accelerated disease model [Figure 1]. The targeted PV was corrected in 32% of transcripts. At this level of editing, the majority of cardiomyocytes would continue to express the dominant HCM allele, yet surprisingly this moderate degree of transcript correction fully normalized the cardiac phenotype. Bystander editing near the R403Q target, or detectable A to I RNA deaminase activity, was not detected.

The Seidman lab applied a similar dual AAV strategy to correct R403Q variant within the native murine *Myh6* context^[4]^. Using a different ABE variant, the authors demonstrated that intrathoracic delivery of 2.5E13 dual AAV at P10-P13 corrected 81% of LV transcripts, resulting in no detectable morphological differences between treated *Myh6*^*R403Q/+*^ and wild-type mice for 32-34 weeks. Bystander editing that changes the encoded protein, was detected in 5% of *Myh6* transcripts. Minimal on-target editing was detected in genomic DNA from non-cardiac tissues. DNA sequencing of left ventricular genomic DNA at experimentally determined candidate off-target sites revealed up to 8% off-target editing. Considering that cardiomyocytes constitute approximately one-third of the left ventricular myocardium, ~25% of cardiomyocytes may have this off-target edit. The authors also tested an alternative genome modification strategy, the inactivation of the mutant R403Q allele by introducing indels using *S. aureus* Cas9. Efficient targeting of the mutant allele was achieved, but significant and dose-dependent inactivation of the wild-type allele also occurred, associated with dose-dependent cardiac dysfunction. Although a dose was identified that ameliorated HCM without causing cardiac dysfunction, the therapeutic index was low. This result suggests that allele-selective gene inactivation would be difficult to translate.

These studies advance the field by demonstrating that highly efficient and precise base editing can be achieved *in vivo* in postnatal cardiomyocytes and deployed to treat inherited heart conditions caused by dominant missense mutations. This strategy will be applicable to conditions caused by C↔T or G↔A single nucleotide variants within sequences amenable to precise base editing. Compared to gene replacement by episomal AAV-mediated expression, genome editing may avoid cargo limitations that preclude the expression of large gene products. Gene editing can treat dominant PVs, such as *MYH7*^*R403Q*^, unlike gene replacement strategies more typically pursued in AAV gene therapy, which are better suited to correcting insufficient gene expression. Gene editing is also invulnerable to loss of AAV episomes, which may lead to waning efficacy of traditional AAV gene therapy over many years.

On the other hand, genome editing comes with its own challenges and limitations. Base editing, like other allele-specific therapies, will require a distinct therapeutic product for each PV. There are over 1,500 PVs known to cause HCM, and developing a separate product for each will be a daunting task. Prime editing^[8]^, an alternative genome editing technology, may offer an advantage in that one editing product can modify thousands of bases and thereby address many different variants. Nevertheless, base editing of more prevalent variants could benefit thousands of patients. For example, Chai *et al.* estimated that over 25,000 patients worldwide may carry the *MYH7*^*R403Q*^ variant alone^[3]^. Undesired editing is another risk of genome editing, particularly when genome-modifying enzymes are expressed using AAV, which will persist for many years. On-target efficiency needs to be balanced with bystander and off-target editing. For instance, due to the selection of different ABE editors (and different target sequences), Chai *et al.* did not detect bystander editing (off-target editing was not measured *in vivo*) but only achieved 32% on-target editing^[3]^. In contrast, Reichart *et al.* achieved 81% on-target editing but with up to 5% bystander and potentially 25% off-target editing^[4]^. Given that the longest duration studied, ~8 months in Reichart *et al.*, is only a small fraction of a human lifespan, and the likelihood that off-target edits will accumulate over time, a better understanding of potential genome editing toxicities is critical to inform clinical translation of this technology^[4]^. These challenges will be addressed by the continued development of editors with improved efficiency and precision, and by technologies that limit the strength or duration of editor expression.

The exciting advances made by Chai *et al.* and Reichart *et al.* towards a novel therapeutic approach to HCM lead to important follow-up questions^[3,4]^. These studies used base editing to prevent the development of HCM. However, many patients, including those with the most pressing need for novel therapies, have established diseases. Moreover, many HCM PVs have incomplete penetrance and variable expressivity. As a result, it is often best to treat patients based on their clinical indications. To be deployed in these scenarios, it will be important to demonstrate that correcting an HCM mutation can reverse or arrest the progression of established disease. These studies used an unconventional delivery route, intrathoracic injection, and targeted perinatal cardiomyocytes, which might be more amenable to editing. Another study from the Olson group^[9]^ documented efficient base editing in adult cardiomyocytes, albeit with direct myocardial injection of high viral doses. It will be important to determine if delivery of base editors to adults using translatable routes of administration will achieve the same efficacy at tolerable AAV doses.

The studies by Chai *et al.* and Reichart *et al.* are beautiful proof-of-concept demonstrations of the power of modern genome editing tools, which hold the potential to precisely modify the genome and free patients from the tyranny of the genetic code^[3,4]^.

## Figures and Tables

**Figure 1.**
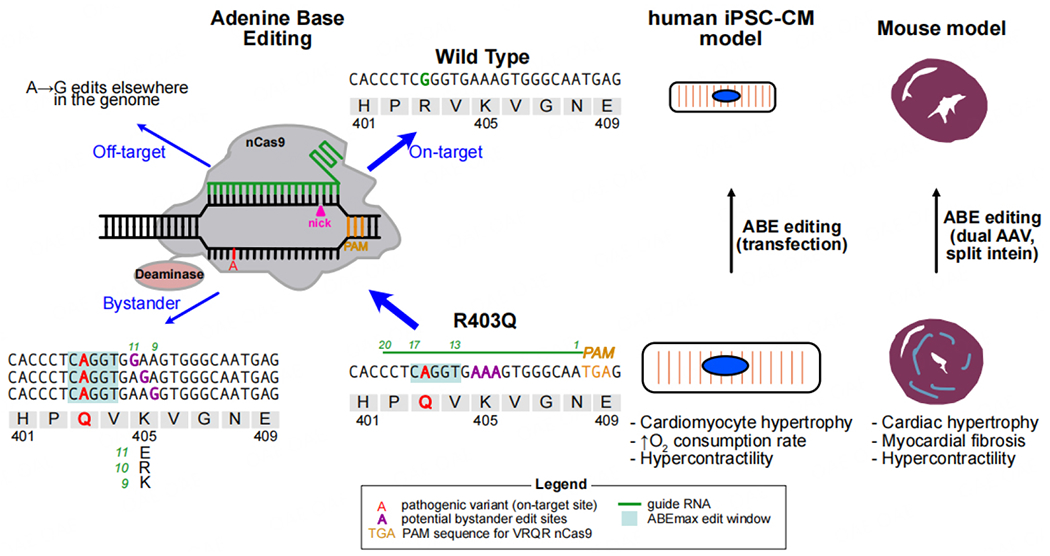
Base editing for HCM caused by the *MYH7*^*R403Q*^ pathogenic variant. The sequences of wild-type and R403Q MYH7 near the pathogenic variant are shown, along with a schematic of the adenine base editing system. The base editor is targeted to the R403Q allele by a specific guide RNA (green line). Three types of edits are possible: 1. on-target reversion of the pathogenic “A” (red) to “G” (green), converting the HCM allele into a normal allele; 2. Bystander editing, where another adenine near the targeted residue, most often within an “editing window” (light blue) is converted to G (purple), introducing additional missense changes into the encoded protein; and 3. Off-target editing. This occurs when the guide RNA targets the base editor to other regions in the genome. The relative frequency of on-target vs bystander and off-target editing is a major determinant of benefit vs. risk of base editing. Successful editing ameliorated pathological HCM phenotypes in human iPSC-CM and mouse models of HCM caused by *MYH7*^*R403Q*^.

## Data Availability

Not applicable.
